# Cervical Muscle Activation Due to an Applied Force in Response to Different Types of Acoustic Warnings

**DOI:** 10.1007/s10439-021-02757-4

**Published:** 2021-03-25

**Authors:** Mohammad Homayounpour, Nicholas G. Gomez, Anita N. Vasavada, Andrew S. Merryweather

**Affiliations:** 1 Department of Mechanical Engineering, University of Utah, Salt Lake City, UT USA; 2 Gene and Linda Voiland School of Chemical and Bioengineering, Washington State University, Pullman, WA 99164-6515 USA; 3 Department of Integrative Physiology and Neuroscience, Washington State University, Pullman, WA 99164 USA; 4 Washington Center for Muscle Biology, Washington State University, Pullman, WA 99164 USA

**Keywords:** Whiplash, Neck muscle, Directional acoustic warning, mTBI, Muscle-activation, Startle

## Abstract

Mild traumatic brain injury (mTBI) and whiplash-associated disorder are the most common head and neck injuries and result from a sudden head or body acceleration. The head and neck injury potential is correlated with the awareness, level of muscle activation, and posture changes at the time of the perturbation. Environmental acoustic stimuli or a warning system can influence muscle activation and posture during a head perturbation. In this study, different acoustic stimuli, including Non-Directional, Directional, and Startle, were provided 1000 ms before a head impact, and the amplitude and timing of cervical muscle electromyographic (EMG) data were characterized based on the type of warning. The startle warning resulted in 49% faster and 80% greater EMG amplitude compared to the Directional and Non-Directional warnings after warning and before the impact. The post-impact peak EMG amplitudes in Unwarned trials were lower by 18 and 21% in the retraction and rebound muscle groups, respectively, compared to any of the warned conditions. When there was no warning before the impact, the retraction and rebound muscle groups also reached their maximum activation 38 and 54 ms sooner, respectively, compared to the warned trials. Based on these results, the intensity and complexity of information that a warning sound carries change the muscle response before and after a head impact and has implications for injury potential.

## Introduction

Experimental and simulation studies have investigated the role cervical muscles play in stabilizing the head and neck, as well as how those muscles might become injured.^33^,^40^ The muscles of the head–neck complex have a central role in the abatement of higher head accelerations and are a potential site of injury and pain.^48^ Mild traumatic brain injuries (mTBI) and whiplash-associated disorders (WAD) are the most common head and neck injuries resulting from either direct head impacts or body accelerations. It has been estimated that 3.8 million sports-related mTBI occur in the US annually.^20^ WAD is the result of forceful, rapid back-and-forth movement of the neck and affects 4 in 1000 persons annually in the United States.^52^ WAD is often the result of motor-vehicle collisions as well as sports injuries.^19^ Clinical evidence of muscular damage resulting from whiplash is potentially debilitating pain.^14^ Researchers have speculated that injury occurs due to eccentric loading or forceful lengthening of contracted muscles. Muscle injuries, specifically contraction-induced strains, are a function of pre-activation, the magnitude of strain, and initial muscle length.^31^ All of these factors are correlated with the loss of contractile force. For a given strain, increased muscle activation levels may result in greater muscle damage, and severity is dictated by the amount of force applied.^36^ Direct muscle injury may not be responsible for chronic whiplash pain and mTBI, however it has been suggested that muscles likely play an indirect role in how pain presents from injuries to other structures.^48^ In events that may result in WAD, such as automobile collisions and impacts to the head during sports, several studies have indicated that there is a neuromechanical delay between cervical muscle activation and head movement.^15^,^33^,^40^,^45^

Cervical muscles act to stabilize the head. Researchers have suggested that positioning the head toward the direction of an impending impact and tensing the neck muscles are effective techniques to decrease the linear and angular velocity of the head following an impact.^16^,^38^,^42^ However, the significance of this effect has only generally been shown in low-severity impacts and the scalability of these results to concussive-level impacts is still unclear.^26^ In addition to physical preparations before a perturbation, awareness of the perturbation may produce anticipation and affect a subject’s kinematics response.^45^ Outside of conscious effort, cervical muscle activation can be manipulated by taking advantage of the acoustic startle response (ASR). Startle varies with sound intensity to an abrupt, intense (> 90 dB) auditory stimulus.^24^,^41^ Startle responses recruit more muscles and present with larger EMG amplitudes when participants are prepared compared to conditions when the stimulus was delivered unexpectedly.^51^ Factors that increase EMG amplitude and probability of an ASR are lower stimulus rise time, higher intensity, and wider bandwidth.^9^

Whether during an automobile collision or an impact while playing sports, awareness of impending contact changes the risk of injury and the head’s kinematic response.^1^,^12^,^26^^–^28,30,45 In football, the lack of awareness and poor posture are reported as contributing factors in concussion.^29^ There are efforts to instrument helmets with antennas to predict and warn players before a severe collision to help them correct their posture or clench cervical muscles before the injurious perturbation.^32^,^37^ Scenarios that can result in a neck injury can also have both audible and visual cues present,^37^ causing either an involuntary or voluntary muscular response. The level of muscle activity and resulting kinematic response from a whiplash-like perturbation are different depending on the type of *a priori* knowledge provided to participants. Though these prior studies have investigated simulated impacts where warnings are present, there is still only a limited amount of information regarding how the magnitude and timing of muscle activity may change in response to varying levels of information.

In this study, we evaluated the effect of different types of acoustic warnings as well as the effect of head and neck posture on the timing and magnitude of cervical muscular response before and after a head impact. Our outcomes were EMG activity as a function of warnings that resulted in either an involuntary muscular response (Startle) or a voluntary muscular response with and without postural change (Directional and Non-Directional, respectively). All stimuli played 1000 ms before the impact, and Directional warnings notified the participants about the impact’s direction. We hypothesized that increasing the sound intensity (Startle vs Non-Directional and Directional) will decrease the EMG onset time (*T*_(Pre-Imp-Onset)_) for different types of warnings before a random direction impending impact. Furthermore, adding information regarding the impending impact’s direction would also reduce *T*_(Pre-Imp-Onset)_ (Directional vs Non-Directional). We also investigated the time (*T*_(Pre-Imp-Max)_) and magnitude (EMG_(Pre-Imp-Max)_) of maximum EMG following the acoustic warnings as that time interval has not often been quantified in the literature and can vary depending on the characteristics of the acoustic stimulus. We hypothesized that all warnings would increase the maximum EMG activation (EMG_(Post-Imp-Max_), and decrease the time to peak EMG activity (*T*_(Post-Imp-Max)_) after the head impact.

## Materials and Methods

### Participants

Ten male participants (age 26.2 ± 3.1 years, height 179.8 ± 5.3 cm, and weight 73.6 ± 7.6 kg) were recruited under the University of Utah Internal Review Board (IRB: 94138) protocol. Participants were included if they had no history of concussions or neck injuries.

### Instrumentation

**Figure 1:**
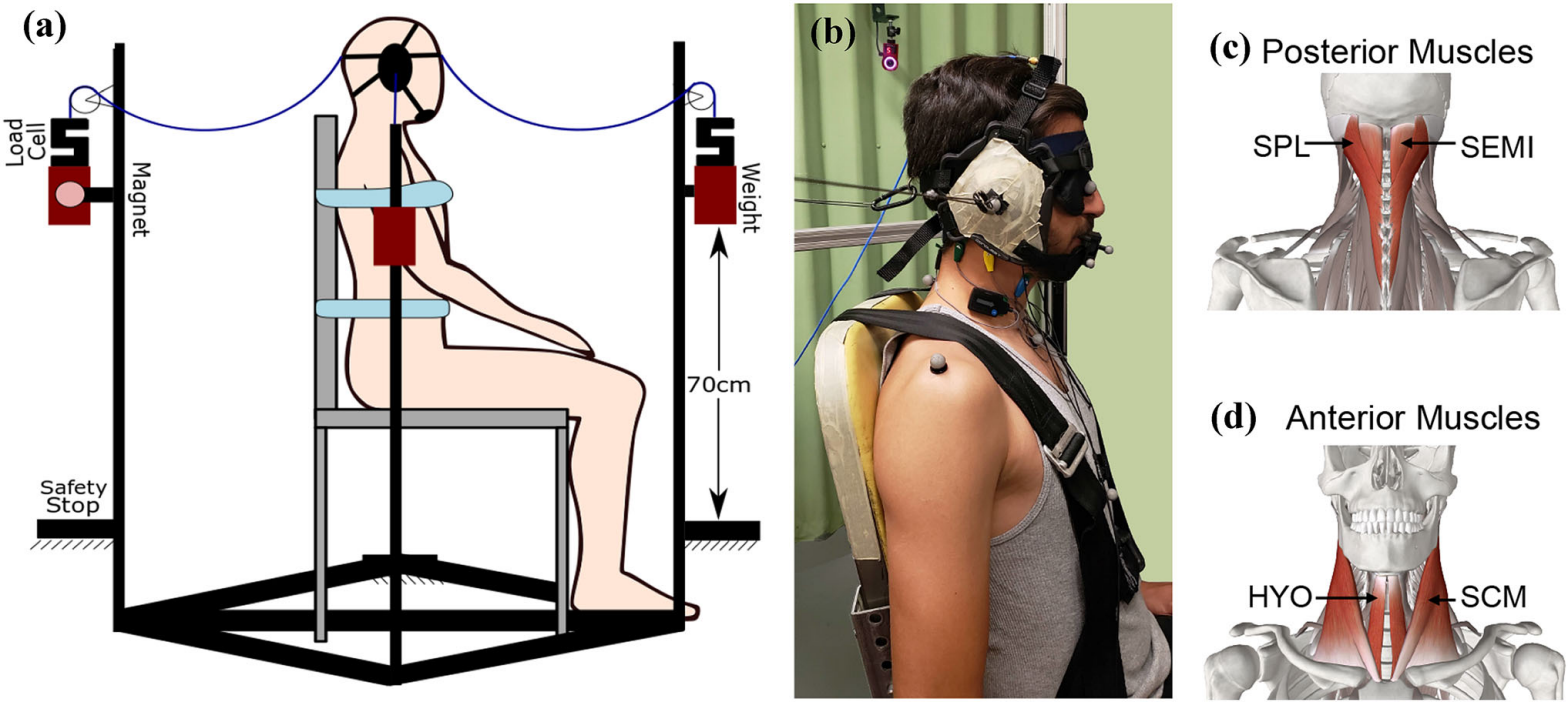
(a) Schematic of the testbed. The testbed includes four weights, allowing for impulsive loads to be applied in four different directions without modifying the experimental setup. We simulated the impact with a 1.2 kg weight, attached to the head gear with Kevlar cable. The Kevlar cable has slack to allow the mass to free fall on the linear guide for 60 cm and then pull the head. A safety stop was placed 10 cm after the end of the string to make sure the neck would not be overextended. Participants were strapped to the chair to minimize trunk movement. We asked participants to place their hands on their laps. (b) Participant in the experimental setup: headgear sized and sewn for each subject before the test to reduce slipping. We used a blindfold and noise isolation earbuds to prevent audio or visual cues other than the controlled warning sounds, prior to the impact. (c) EMG sensors were placed bilaterally over the posterior, semispinalis capitis (SEMI) and splenius capitis (SPL), and (d) anterior, sternocleidomastoid (SCM) and hyoid (HYO), muscles.

A headgear (ASICS Adult Conquest Wrestling Headgear) was custom-fit to each participant for administering safe impacts. Muscle activations were measured using eight surface electromyography electrodes (EMG) (Delsys Trigno wireless EMG, MA, USA). EMG sensors were placed bilaterally over the sternocleidomastoid (SCM), hyoid (HYO) and semispinalis capitis (SEMI), and splenius capitis (SPL), muscles as depicted in Fig. 1. The EMG data were collected (EMGWork, Delsys) with a synchronization trigger. Trigno Snap lead (1926 Hz), Avanti (1926 Hz), and Quattro (2222 Hz) sensors were used to measure muscle activations. The data were filtered at 20–450 Hz before being digitized. All EMG data were resampled to 1926 Hz for analysis. Hypoallergenic medical tape secured the sensors and wires on the neck to minimize noise (movement artifact) during the head’s ballistic movement.

An optical motion capture system captured the kinematics of the head and neck. This system consisted of 9 OptiTrack cameras (100fps, Flex 3, NaturalPoint, Corvallis, OR). Reflective markers were placed on both acromioclavicular joints, the jugular notch, the xiphoid process, and the C7 spinous process. To provide redundant tracking of the head in the event of marker occlusion, two additional markers were placed on the nose, and three on a mouthguard fixed to the upper jaw. Two markers were placed on the headgear’s earpieces and one on each pulley to locate the applied forces’ exact direction and location.

Head and neck motion were isolated through the use of a high-back chair with a four-point restraint. We attached four cables to the headgear, one in front, back, right, and left. These cables were attached to a 1.2 kg mass on a linear rail and held in place using an electromagnet. After releasing the mass from the magnet, there was 60 cm slack on the cable, which allowed the mass to free-fall and then applied an impulse to the head. Friction on the rails was negligible. Load cells (50lb, S-Type, PCB, NY) were used in series with the cable and the corresponding mass to measure the impact force. The load cell data were acquired with a NI 9237 (National Instruments Corporation, TX) Strain/Bridge input module with a 2000 Hz sampling rate.

Participants wore earbuds (ER3XR, ETYMOTIC, IL) to deliver the acoustic warnings. These earbuds were capable of playing sounds up to 120 dB verified by sound pressure level meter and coupler (Model 831, Larson Davis, NY, USA, IEC 126 2CC Coupler, G.R.A.S, Denmark). A sound amplifier was used to amplify the output sound from the computer to the earbuds. We used a relay to avoid any inconsistent delay from the computer’s soundcard. The noise isolation with the earbuds and the headband’s earpieces were above ambient noise levels, minimizing external audible sources’ influence. We also used a blindfold to ensure that the participant would not have any visual cues about the incoming impact.

A NI 9264 Analog output module was used to control electromagnets, sound relays, synchronize the EMG system, and turn on an active marker for the motion capture synchronization. A NI cDAQ 9189 was used to synchronize and collect data from the NI 9264, NI 9233, and NI 9237 modules. LabVIEW (National Instruments Corporation, TX) software was used to control all the modules, data collection, and synchronization between all modules. The timing error between all sources was less than 1 ms.

### Warnings

Three different types of warnings played from the earbuds. A sound of 72 dB within the spectral range of 2–20 kHz was played for 500 ms for *Directional* trials, starting 1000 ms before the impact. A train whistle sound, back sound, was played to indicate an impending impact from the back (pulling the head to front). An airhorn sound, the front sound, was played to indicate an impending impact from the front (pulling the head to the back). A sound was played exclusively in either the right or left earbud to indicate an impending impact to either the right (pull to left) or left (pull to right) side of the head, respectively. The participant was asked to move toward the direction of the sound, against the direction of the pull force, as soon as the sound was heard for Directional warnings. Appropriate movement responses were verified following data collection by evaluating the motion capture data. Trials were discarded if the participant moved in the wrong direction or could not decide appropriately before the impact. In *Non-Directional* trials, a buzzer sound at 72 dB was played starting 1000 ms before the impact for 500 ms. This sound was not associated with any impact direction, and the impact direction after this sound was unknown to the participant. The participants were instructed to clench all of their cervical muscles for this condition isometrically. In *Startle* trials, each one of the 5 sounds for Directional and Non-Directional warnings were amplified to 115 dB and played for 500 ms in 5 different startle trials. No instructions were provided for this condition as the stimulus was capable of generating an involuntary muscular response. Startle trials were discarded if no muscle activation, in any muscle, were present within the first 100 ms after starting the sound. In the *Unwarned* (control) condition, no warning was given prior to the impact. The timing and direction of the impact were unknown to the participant. Overall, 17% of the trials were omitted due to an inappropriate response from the participants.

### Protocol

After applying the instrumentation to the participant, a maximum voluntary contraction (MVC) test was performed. Cables were fixed to the participant’s headband while maintaining a self-selected neutral head and neck posture. Directional stimuli were played for four principal directions, and the participants were asked to exert their maximum force toward the direction of the sound for four seconds. Participants were asked for the Non-Directional stimulus to perform maximum neck clenching in a neutral posture for four seconds. During the MVC trials, verbal motivation was given to participants.^44^ The MVC was completed two times prior to impacts and one time following the impact trials at the end of the test.

Following the MVC trials, Non-Directional and Directional warnings were played for the participants at least three times before delivering any impacts to help with acclimation to the warning sounds. Participants underwent response training with no applied impacts until they performed as instructed for all the Non-Directional and Directional warnings. After that, participants experienced five training runs where impacts were applied after the warning. In these trials, the participant was told about the impacts’ direction and timing, and following their confirmation, the sound was played, and perturbation was applied. These training trials were not reported due to differences in the type of response, related to single reaction time vs. choice reaction time, and possible habituation effects.^6^,^8^,^46^

After 5 training impacts, 15 test trials that included only Non-Directional and Directional impacts were applied randomly in four directions. For these trials, the acoustic warning was the only cue provided to the participant. Immediately after the first 15 test impacts, without notifying the participant, there was a mixture of 30 impacts that included Directional, Non-Directional, Startle, and Unwarned trials for a total of 45 test trials. These 30 impacts consisted of 5 Startles, 12 Directional, 3 Non-Directional warnings, and 10 Unwarned delivered in random order. Including the five training trials, the Startles were placed at trials 23, 30, 36, 42, and 48 to avoid habituation. The participant experienced all 45 test trials during the same session, and there were random 20 to 45 seconds delays between each impact.

### EMG Data Analysis

All EMG data were high-pass filtered after data acquisition at 30 Hz to remove motion artifacts before calculating the root-mean-squared (RMS) values using a zero-phase 50 ms moving window. Trials with noisy EMG data (*N* = 6) were dismissed as the results were deemed unreliable and usually resulted from a poor connection between the sensor and the skin or if the headgear contacted the sensor. The maximum RMS value for each muscle was computed within the 1–3 seconds of the MVC trials. The EMG activation was then normalized based on the MVC value. The baseline muscle activation for a trial was defined as the minimum RMS value for that muscle after the warning.

**Figure 2:**
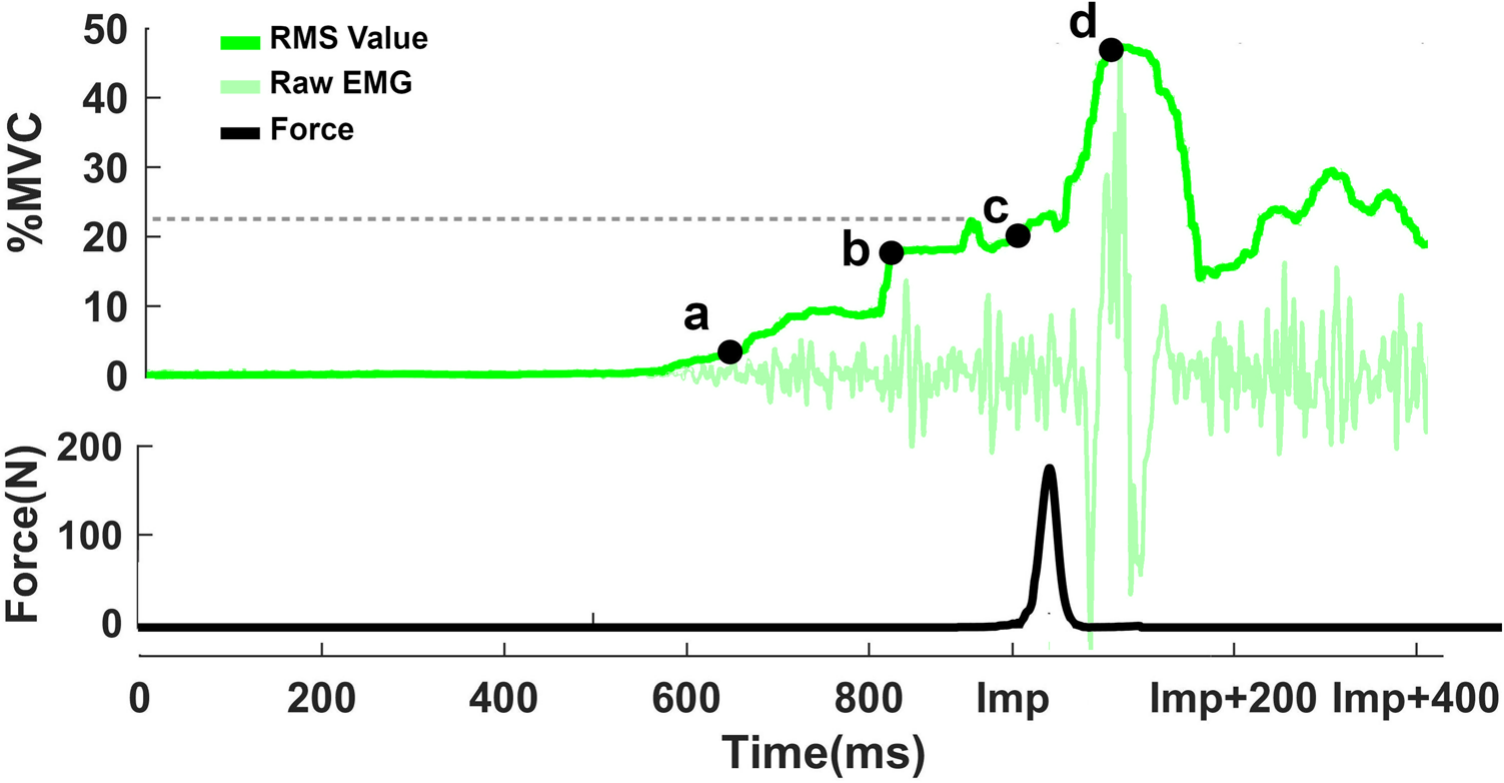
A sample of EMG amplitude for the SCM muscle and the force profile in that trial. The warning started playing at Time = 0 and impact happened at the time = Imp. (a) *T*_(Pre-Imp-Onset)_ represented the first time that the value of muscle activation reached 3%MVC. The dashed line indicates the absolute maximum value after the warning and before the impact for that test trial for the SCM muscle. (b) *T*_(Pre-Imp-Max)_ and EMG_(Pre-Imp-Max)_ defined when the EMG amplitude reached 70% of its maximum amplitude before the impact in that trial, (c) EMG_(Imp)_ represented EMG amplitude at the onset of the force, and (d) T_(Post-Imp-Max)_ and EMG_(Post-Imp-Max)_ showed the peak EMG activation after the impact. Points a and b, T_(Pre-Imp-Onset)_ and *T*_(Pre-Imp-Max)_, times are reported based on the time of warning, *t* = 0. For point d, *T*_(Post-Imp-Max)_, time was reported based on impact time *t* = Imp.

In Fig. 2, *t* = 0 is the time that the sound started playing. The load cell data were used to measure force and the exact impact time. The force onset was defined when the force reached 10 N. Impact time varied due to the head’s position in the testbed, and onset forces happened on average at 998 ± 25 ms after the warning. To precisely synchronize the post-impact EMG events, the force onset time was used as the second time reference point, *t* = Imp.

*T*_(Pre-Imp-Onset)_, point “*a*” in Fig. 2, was defined as the first time each muscle amplitude reached more than 3%MVC after removing the baseline. This onset time was visually evaluated to eliminate artifacts. After calculating the *T*_(Pre-Imp-Onset)_ for all the eight muscles, the average value of the two shortest times was reported as the *T*_(Pre-Imp-Onset)_ of that trial. *T*_(Pre-Imp-Max)_ before the impact, point “*b*” in Figure 2, was defined as the time when an investigated muscle reached 70% of its maximum amplitude in that trial before the impact. This result was only calculated if the maximum muscle activation reached more than 5%MVC in that trial. The median of the eight muscles’ 70% amplitude times was reported as the *T*_(Pre-Imp-Max)_. Then, the eight muscle activation amplitudes at that time were averaged as EMG_(Pre-Imp-Max)_. *T*_(Pre-Imp-Onset)_ and *T*_(Pre-Imp-Max)_ were reported in reference to *t *= 0 within 950 ms after the warning, and the *T*_(Post-Imp-Max)_ was referenced to *t *= Imp. EMG_(Imp)_ is the average level of muscle activations at t= Imp and represents muscle activation levels just before the impact. EMG_(Post-Imp-Max)_ is defined as the mean of the maximum activation for each muscle group within the 400 ms after the impact. *T*_(Post-Imp-Max)_ is the average time that these peaks happened after the impact for the muscle group.

### Statistics

Our Post-Warning EMG outcome variables of interest were *T*_(Pre-Imp-Onset)_, *T*_(Pre-Imp-Max)_, and EMG_(Pre-Imp-Max)_. Our Post-Impact EMG outcome variables of interest were EMG_(Imp)_, EMG_(Post-Imp-Max)_, and *T*_(Post-Imp-Max)_. For post-impact EMG outcomes, the eight muscles monitored during the protocol were combined to make four groups: anterior (bilaterally HYO and SCM), posterior (bilaterally SPL and SEMI), right (R-HYO, R-SCM, R-SEMI, R-SPL), and left (L-HYO, L-SCM, L-SEMI, L-SPL). The values in each group were averaged together. The muscle group antagonist to the direction of pull was referred to as the retraction muscle group, and the muscle group agonist to the direction of pull was referred to as the rebound muscle group (i.e. in the back pull, anterior and posterior muscles are retraction and rebound muscles, respectively). Retraction values refer temporally to EMG information following the head’s initial movement due to an applied load. Rebound values refer to EMG that occurs after the head changes the direction of movement.

Linear mixed models were fit to describe each EMG outcome. Linear mixed models (LMM) are able to treat participants as a random effect, which allows for the results to be generalized to the population of participants and the population of conditions.^3^ Incomplete and unbalanced data can also be used in LMM since information loss due to averaging over observations or participants is avoided.^10^,^23^ Separate models were run for each direction of pull for retraction and rebound muscles. These models had warning types (Directional, Non-Directional, Startle, and Unwarned) defined as the fixed effect, and all models designated participants as a random effect. Model assumptions were validated by examining the normality of residuals.^18^ Pairwise comparisons were performed on the model to check for the significance between the types in the model. Multiple comparisons were controlled using the Benjamini–Hochberg method.^5^ A compensated (scaled) *p*-value of less than 0.05 was considered statistically significant in the linear mixed model in MATLAB 2020 (MathWorks, Natick, MA, USA). The summary of all fits, *F*-test, estimated means, standard errors, and *p*-values are presented in Appendix 1.

## Results

**Figure 3:**
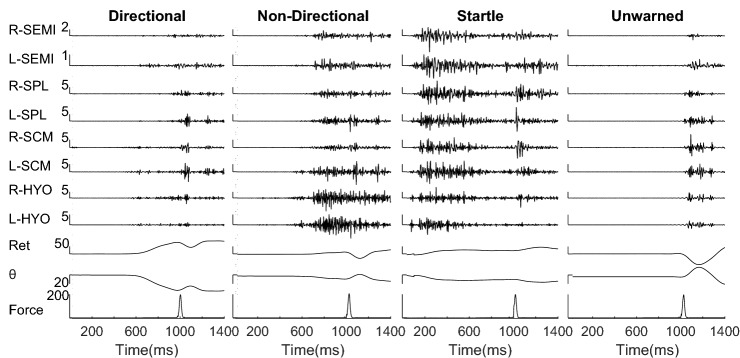
Muscle and kinematic response to different types of warnings and the head impact pulling the head in sagittal extension. Warning started at *t* = 0 and lasted for 500 ms. Right (R-) and left (L-) semispinalis capitis (SEMI), splenius capitis (SPL), sternocleidomastoid (SCM), hyoid (HYO) muscle activations, head retraction (Ret), head rotation (*θ*) and the applied force are presented. The vertical scale bars are aligned to the sound onset and are in the scale of 10^−1^ mV for muscle activations, mm for retraction, degrees for *θ* and *N* for force, respectively. The startle trials started with an early response compared to the Directional and Non-Directional. There is no muscle activation in the Unwarned trial before the impact.

**Figure 4:**
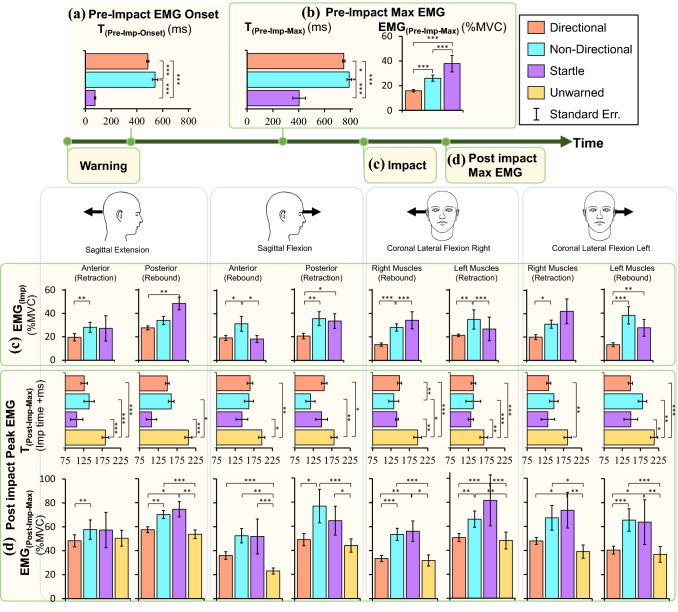
Summary of all muscle activations and their timings based on the type of warnings for the events defined in Fig. 2. (a) EMG onset, *T*_(Pre-Imp-Onset)_, was significantly faster with Startle warning compared to Directional and Non-Directional warnings. (b) EMG_(Pre-Imp-Max)_ and *T*_(Pre-Imp-max)_ were substantially faster and higher with the startle response compared to Directional and Non-Directional warnings. (c) EMG_(Imp)_ based on the type of the warnings, the direction of the impact (sagittal extension, sagittal flexion, coronal lateral flexion to the right and to the left), and muscle groups (anterior, posterior, right, and left muscles). Since the muscle activation before the impact were less than 1% in Unwarned condition, they were not reported before the impact. (d) EMG_(Post-Imp-Max)_ and *T*_(Post-Imp-max)_ represented the amplitude and of the peak muscle activation after the impact. Significance: *p < 0.05, **p < 0.001, ***p < 0.0001.

The average peak head impact force for the 371 trials in which participants exhibited the correct response to the warning stimulus was 179.8 ± 20.6 N (mean ± SE). The average peak linear acceleration due to this impact was 49.75 ± 13.9 ms^−2^. The direction of the warning did not affect the response time in Directional warning, and as a result, the results were combined for all four directions. Figure 3 shows the sampler EMG and the head and neck’s basic kinematic response following a head pull in sagittal extension. The estimated mean for EMG onset, *T*_(Pre-Imp-Onset)_ in Fig. 4a, with Startle response (59 ms) was faster than Directional (483 ms) and Non-Directional (540 ms). Max EMG before impact, EMG_(Pre-Imp-Max)_, was greatest for Startle response (38%MVC) compared to Non-Directional (26%MVC) and Directional (16%) warnings (Fig. 4b). The max EMG before the impact, *T*_(Pre-Imp-Max),_ happened earliest for Startle response (394 ms) compared to Directional (747 ms) and Non-Directional (791 ms) warnings. EMG_(Imp)_ for the Directional warning (20%MVC) was the lowest compared to the Non-Directional (33%MVC) and Startle (32%MVC) conditions (Fig. 4c).

Following the impact, the EMG max time, *T*_(Post-Imp-Max)_, was the highest for the Unwarned trials, which had values of 180 and 203 ms for the retraction and rebound muscles compared to 142 and 152 ms in warned trials, respectively (Fig. 4d). EMG max amplitude after impact, EMG_(Post-Imp-Max)_, was the lowest for Unwarned (46% and 36%MVC) and Directional (50% and 41%MVC) in retraction and rebound muscles compared to Startle (68% and 58%MVC) and Non-Directional (66% and 59%MVC) trials, respectively. Significant differences are reported in Fig. 4 and Table 3 in Appendix 1.

## Discussion

First, we hypothesized that increasing the sound intensity will decrease the EMG onset time (*T*_(Pre-Imp-Onset)_) for different types of warnings in the presence of a random direction impending impact. As we expected, acoustic warnings resulting in an involuntary muscle response (Startle) had a *T*_(Pre-Imp-Onset)_ of 58.7 ms that was at least 425 ms faster than the warnings resulting in a voluntary response (Directional and Non-Directional). Our results align with previous studies.^8^,^24^ Looking specifically at the SCM muscle, the *T*_(Pre-Imp-Onset)_ in our Startle trials measured 72 ± 2.5 ms, which was close to 69 ms reported for habituated conditions from a superimposed sled study^8^ and 65 ms from a study investigating the ASR.^24^ Adding information regarding the direction of an impending impact through a Directional warning reduced the *T*_(Pre-Imp-Onset)_ by 56 ms compared to the Non-Directional, voluntary response. These results align with previous research,^4^,^53^ and support the conclusion that providing directional information decreases choice reaction time.

We considered a participant to be fully prepared for an impending impact when they reached EMG_(Pre-Imp-Max),_ and we sought to quantify *T*_(Pre-Imp-Max)_ for the different types of warnings to establish their respective effectiveness. We expected the Startle response to having the fastest *T*_(Pre-Imp-Max)_ and the largest EMG_(Pre-Imp-Max),_ and our results confirmed that expectation. The Startle response resulted in a *T*_(Pre-Imp-Max)_ 480 ms faster than Non-Directional warning and 394 ms faster than the Directional warning. For the EMG_(Pre-Imp-Max)_, the Startle response was 133% greater than the Non-Directional warning and 58% greater than the Directional warning. After reaching EMG_(Pre-Imp-Max)_, there was an observed decay in EMG amplitude for the Startle response. This decay occurred over 600 ms and resulted in an EMG amplitude that was 46% of the EMG_(Pre-Imp-Max)_ value at the time of impact EMG_(Imp)._ The EMG_(Imp)_ following a startle stimulus was not significantly different from the EMG_(Imp)_ observed for the Non-Directional warning. Our results indicate that Startle responses result in faster *T*_(Pre-Imp-Max)_ and greater EMG_(Pre-Imp-Max),_ but the involuntary muscular responses were diminished after 600 ms, and the response 1000 ms after the stimulus was most likely a voluntary response. The EMG_(Imp)_ for Non-Directional and Directional warnings, on average, did not exhibit a decay compared to EMG_(Pre-Imp-Max)_ but instead stayed the same or slightly increased before the impact.

We hypothesized that all warnings would increase the EMG_(Post-Imp-Max)_ and decrease the *T*_(Post-Imp-Max)_ for the data collected following random direction impacts to the head compared to the Unwarned conditions. In both Non-Directional and Startle warning, the EMG_(Post-Imp-Max)_ for the retraction and rebound muscles were higher in amplitude by 47 and 61%, respectively, compared to Unwarned trials. Although the EMG_(Post-Imp-Max)_ for the Directional warning was 9 and 14% greater in the retraction and rebound muscles compared to the Unwarned condition, this difference was not significant for any of the four directions of impact. Muscle activation in the Unwarned condition reached *T*_(Post-Imp-Max)_ 38 and 50 ms later than all the warned conditions in retraction and rebound muscles. These findings are consistent with previous investigations documenting that increased joint stiffness at the time of impact is associated with a greater reflex amplitude and shorter latency.^2^,^17^,^21^,^49^ Alsalaheen *et al*.^1^ also reported an increase in EMG_(Post-Imp-Max)_ and a reduction in *T*_(Post-Imp-Max)_ for the SCM muscle when participants exhibited a forceful muscle contraction in response to an anticipated head impact compared to the condition where there was no muscle activation, and the impact was unexpected.

Whether the participant was alerted or surprised during a perturbation has been investigated as a contributor to whiplash injuries.^39^,^45^ What has not been investigated thoroughly is the absolute soonest one can expect a muscular response in the presence of either startle or non-startling warnings. We investigated the lower 95% confidence interval time value for the onset of muscle activation, T(LCI-Onset), for all stimuli to answer that question. The Startle stimuli, Non-Directional, and Directional responses had *T*_(LCI-Onset)_ of 29, 441, and 390 ms after the acoustic warning, respectively. Since it has also been suggested that the peak head accelerations are the contributing factors in head and neck injuries,^35^,^50^ knowledge of the timing regarding muscle activations is important as muscle activation may affect the injury risk if it happens before the acceleration peaks. Examples include motor vehicle collisions, where peak accelerations occur within 150 ms of initial vehicle contact, or in contact sports where peak accelerations can occur within 15 ms of contact to the head.^13^,^43^ In both these instances, the mentioned acoustic warnings have to be given to the participant, *T*_(LCI-Onset)_ milliseconds, before peak accelerations to have any kinematic effect on the injury risk. We acknowledge that there could be some other neurological effect due to the acoustic stimulus within the *T*_(LCI-Onset)_ and before a perturbation, which may alter the EMG response, but we did not test for that in this study.

*T*_(LCI-Onset)_ informs us as to whether or not a warning has the potential to influence head kinematics following an impact to the head or acceleration to the body. Higher muscle activation is correlated to more reduction in head linear and angular velocity.^49^ In the scenario where a warning is desired as a protective mechanism, the participant was called “prepared” when EMG_(Pre-Imp-Max)_ was reached after the warning. We characterized this preparation time, *T*_(Pre-Imp-Max),_ as a function of different acoustic warnings. Our results indicate that the Startle response, by the highest EMG_(Pre-Imp-Max)_, has the greatest potential to influence head kinematics. Also, the shortest *T*_(Pre-Imp-Max)_ latency may have application in a warning system application to reduce the number of false alarms in the collision prediction algorithm before an imminent head impact or a collision. We have identified that a potential disadvantage of relying on a startle response is that there is only a 66% probability of achieving a startle response in the SCM muscle in a laboratory setup with 124 dB ASR.^11^ Our results confirm this limitation as we recorded a 58% startle probability in our participants with 115 dB ASR. However, despite not achieving a 100% startle response success rate, our results did show that the response to the startle stimuli is still potentially the most effective warning as our “failed startle responses” still had a higher EMG peak and shorter onset time compared to the Directional/Non-Direction trials (*T*_(Pre-Imp-Onset)_ = 304 ms, EMG_(Pre-Imp-Max)_ = 23%MVC, and *T*_(Pre-Imp-Max)_ = 637 ms).

We have shown that for a given applied force, the EMG_(Post-Imp-Max)_ is influenced by the EMG_(Imp)_, which is influenced by an acoustic stimulus. Although greater muscle activation before an impact has been correlated with a lower level of head injury potential, these higher muscle activations may increase muscular injury potential during an impact.^36^ We measured lower EMG_(Post-Imp-Max)_ in Unwarned conditions, which may imply a lower risk of injury to the neck muscles. However, this reduced risk only applies to low-level impacts that do not result in the cervical spine exceeding its normal range of motion. If the cervical muscles are relaxed in the presence of a large impact, other anatomical structures such as the anterior longitudinal ligament, cervical discs, and facet joint capsules may experience damage instead of the cervical muscles. This scenario should be avoided as these structures are less likely to heal than injured muscles. Our Directional warnings sought to test one potential injury mitigation strategy apart from isometric cervical muscle contraction. By positioning the head toward the impact’s direction, against the pull, the participants achieved several changes that could influence their injury potential: they increased the cervical range of motion their head could travel that reduced the risk of overextension; they shortened their retraction muscles, which may reduce the strain injury,^22^ and changed the position of the center of rotation of their head.^25^ Due to these postural changes resulting from a Directional warning, the EMG_(Post-Imp-Max)_ was the lowest compared to the Startle and Non-Directional trials, while the *T*_(Post-Imp-Max)_ was similar to the other warned trials. These results indicate that for low-level loads, postural changes may be beneficial as they reduce the risk of muscular strain injury as well as injuries to other cervical soft tissues^34^ that result from head dynamics. Future studies should investigate the tradeoff between the kinematic response and muscle activation level to find an optimum response that mitigates injuries to all structures in the neck.

A few limitations of this study should be noted. We used a head impactor to create the whiplash-like perturbation and applied sudden forces directly to the head instead of using a sled testbed. This method has often not been used in the literature, as most WAD events are reported from motor-vehicle collisions. Our time values may differ from published literature using a sled testbed as the trunk movement in a sled test may trigger additional tactile stimuli before the head’s movement, 39 ms,^7^ compared to 10 ms delay when using a head impactor. In a head impact test, vestibular and trigeminal reflexes may become activated earlier, whereas, in sled tests, other cutaneous reflexes may be contributing, especially in Unwarned trials. However, the EMG results will likely not change significantly between the two test fixtures in warned trials since participants are prepared for the perturbation. We also recognize that the head’s kinematic response can be different in a sled test compared to a direct head impact, though this likely only influences values following motion and not the pre-impact muscle activations. Regarding participants, we only tested male participants. There have been documented gender differences in reaction time and risk of experiencing a neck injury^7^,^12^,^24^ that we hope to address in future studies. To test for habituation, we averaged the RMS EMG values in the first 500 ms for each of the eight muscles. We then averaged the mean of each muscle together for each startle trial to generate a single mean. To test for significant difference between the first trial and any of the other trials, we fitted a LMM to this outcome and we designated the trial number as the fixed factor and we assigned the participant number as the random factor. The results did not show any significance (*p* > 0.159) and as a result, we did not observe habituation from the study protocol. The lack of habituation observed in this study for startle responses may be the result of motor readiness.^47^

This research suggests that the startle warning can be a potential candidate as a warning system before an impending head impact as it resulted in the fastest preparation time and greatest peak muscle activation. Future studies are needed to study the effect of applying the impact at the peak muscle activation during a startle response. We also documented that postural changes resulted in the least amount of muscle activity in the warned condition and faster response compared to the unwarned condition. Additional studies to evaluate how these changes may reduce the risk of injury to muscles and other structures are warranted. Future work is needed to investigate the head’s kinematic response as a function of different muscle activation levels, types of warning, and gender differences.

## Appendix: Result of the Statistical Models

See Tables 1, 2, 3.

**Table 1:** Estimated mean (Est. mean) and Standard error (SE) estimated from the linear mixed models.

	*T*_(Pre-Imp-Onset)_(ms)		EMG_(Pre-Imp-Onset)_(%MVC)		*T*_(Pre-Imp-Max)_(ms)	
	Est. mean	SE	Est. mean	SE	Est. mean	SE
F_Stat (P_Val)	342.5 (< 0.001)		49.6 (< 0.001)		152.7 (< 0.001)	
Startle (Int.)	58.7	19.7	37.6	3.0	394.3	23.4
Non-Directional	539.6	19.8	25.5	2.7	791.6	24.6
Directional	483.4	17.2	16.1	2.3	747.5	21.3

Time of the muscle onset, *T*_(Pre-Imp-Onset),_ and time, *T*_(Pre-Imp-Max)_, and amplitude, EMG_(Pre-Imp-Max)_, of the max muscle activation due to the warning are reported in the columns

**Table 2:** Estimated mean (Est. mean) and Standard Errors (SE) derived from the linear mixed model with the F_statistics (F_Stats) of the model and the corresponding p value.

	EMG amplitude at Imp onset EMG_(Imp)_ (%MVC)				Post-impact Max EMG T_(Post-Imp-Max)_ (ms)				Post-impact Max EMG EMG_(Post-Imp-Max)_ (%MVC)			
	Retraction muscles		Rebound muscles		Retraction muscles		Rebound muscles		Retraction muscles		Rebound muscles	
	Est. mean	SE	Est. mean	SE	Est. mean	SE	Est. mean	SE	Est. mean	SE	Est. mean	SE
Sag. Ext. (*N* = 94)												
F_Stat (P_vale)	44.9 (< 0.001)		35.8 (< 0.001)		15.4 (< 0.001)		8.9 (< 0.001)		2.6 (0.056)		7.8 (< 0.001)	
Unwarned (Int.)	0.0	2.2	1.9	4.3	183.8	7.2	205.4	10.3	50.2	4.1	53.6	10.3
Startle	26.3	3.9	45.9	5.8	106.1	16.1	106.8	23.7	53.8	5.1	71.4	5.9
Non-directional	28.0	2.8	34.2	4.0	139.7	11.5	159.5	16.3	57.2	3.6	69.7	4.0
Directional	19.9	2.3	27.6	3.3	126.3	9.4	148.9	13.5	48.0	3.0	56.1	3.3
Sag. Flex. (*N* = 79)												
F_Stat	43.1 (< 0.001)		27.5 (< 0.001)		5.1 (0.003)		5.3 (0.002)		6.7 (< 0.001)		7.5 (< 0.001)	
Unwarned (Int.)	0.0	2.4	0.8	1.8	181.6	10.2	197.5	11.9	44.7	9.4	23.4	4.3
Startle	33.4	4.1	18.3	3.9	149.1	16.6	155.1	14.7	63.8	6.9	51.0	7.3
Non-directional	35.1	4.4	31.4	4.4	118.9	17.8	170.2	16.7	74.5	7.4	50.3	8.2
Directional	21.3	2.5	19.2	2.5	155.9	10.3	165.3	9.2	54.0	4.3	36.9	4.5
Coronal Lat. Right. (*N* = 102)												
F_Stat (P_Val)	24.2 (< 0.001)		31.2 (< 0.001)		13.4 (< 0.001)		16.1 (< 0.001)		10.1 (< 0.001)		12.7 (< 0.001)	
Unwarned (Int.)	0.0	4.0	0.9	2.8	186.3	10.6	212.9	13.2	48.2	8.5	31.8	5.3
Startle	42.6	6.4	32.6	4.7	141.6	14.4	152.3	17.0	80.1	8.0	52.4	7.1
Non-directional	31.1	4.3	28.6	3.2	149.7	9.8	183.0	11.6	65.7	5.4	54.2	4.8
Directional	19.7	3.5	13.4	2.6	136.3	7.9	150.7	9.4	49.2	4.4	32.7	3.9
Coronal Lat. Left. (N = 96)												
F_Stat (P_Val)	27.5 (< 0.001)		25.3 (< 0.001)		3.4 (0.02)		8.8 (< 0.001)		5.3 (0.002)		10.1 (< 0.001)	
Unwarned (Int.)	0.0	3.4	1.4	3.1	167.5	12.1	197.1	13.1	39.3	7.8	36.0	7.5
Startle	29.3	4.9	27.5	4.8	144.6	14.7	152.7	16.8	73.7	9.9	56.9	6.1
Non-directional	34.1	4.6	38.0	4.5	146.4	13.7	141.2	15.8	65.1	9.3	63.6	5.7
Directional	22.2	2.9	13.7	3.0	138.6	9.0	145.9	10.3	48.8	6.1	42.0	3.8

The Unwarned trial was assigned as the intercept for all the models. The data were categorized based on the forced movement of the head, because of the pull in four different directions. The columns show the resulting value for Impact EMG and the Post-Impact Peak EMG values in the retraction muscles and rebound muscles. *N* for each force direction shows the respective sample size

**Table 3:** Table of significance.

	Muscle group	Warning type	Estimated means				Type vs type	*p*-value			
			Back	Front	Right	Left		Back	Front	Right	Left
EMG_(Imp)_ (%MVC)	Retraction	Startle (S)	26.3	33.4	42.6	29.3	SN	0.745	0.801	0.120	0.498
		Non-directional (N)	28.0	35.1	31.1	34.1	SD	0.141	**0.011**	**0.001**	0.166
		Directional (D)	19.9	21.3	19.7	22.2	ND	**0.006**	**0.007**	**0.006**	**0.011**
	Rebound	Startle (S)	45.9	18.3	32.6	27.5	SN	0.100	**0.029**	0.500	0.100
		Non-directional (N)	34.2	31.4	28.6	38.0	SD	**0.006**	0.844	**0.001**	**0.006**
		Directional (D)	27.6	19.2	13.4	13.7	ND	0.128	**0.014**	**0.001**	**0.001**
*T*_(Post-Imp-Max)_ (ms)	Retraction	Unwarned (U)	183.8	181.6	186.3	167.5	US	**0.001**	0.092	**0.007**	0.173
		Startle (S)	106.1	149.1	141.6	144.6	UN	**0.001**	**0.003**	**0.001**	0.179
		Non-directional (N)	139.7	118.9	149.7	146.4	UD	**0.001**	**0.028**	**0.001**	**0.006**
		Directional (D)	126.3	155.9	136.3	138.6	SN	0.090	0.226	0.662	0.923
							SD	0.267	0.749	0.753	0.741
							ND	0.292	0.070	0.151	0.612
	Rebound	Unwarned (U)	205.4	197.5	212.9	197.1	US	**0.001**	**0.012**	**0.002**	**0.019**
		Startle (S)	106.8	155.1	152.3	152.7	UN	**0.014**	0.155	**0.022**	**0.002**
		Non-directional (N)	159.5	170.2	183.0	141.2	UD	**0.001**	**0.003**	**0.001**	**0.001**
		Directional (D)	148.9	165.3	150.7	145.9	SN	0.067	0.560	0.12	0.635
							SD	0.116	0.579	0.923	0.742
							ND	0.580	0.821	**0.003**	0.794
EMG_(Post-Imp-Max)_ (%MVC)	Retraction	Unwarned (U)	50.2	44.7	48.2	31.8	US	0.579	**0.015**	**0.001**	**0.003**
		Startle (S)	53.8	63.8	80.1	52.4	UN	0.092	**0.001**	**0.005**	**0.015**
		Non-directional (N)	57.2	74.5	65.7	54.2	UD	0.566	0.058	0.844	0.173
		Directional (D)	48.0	54.0	49.2	32.7	SN	0.612	0.310	0.120	0.560
							SD	0.320	0.214	**0.001**	**0.015**
							ND	**0.017**	**0.015**	**0.002**	0.090
	Rebound	Unwarned(U)	53.6	23.4	39.3	36.0	US	**0.009**	**0.001**	**0.011**	**0.003**
		Startle (S)	71.4	51.0	73.7	56.9	UN	**0.001**	**0.005**	**0.001**	**0.001**
		Non-Directional (N)	69.7	50.3	65.1	63.6	UD	0.561	**0.010**	0.844	0.158
		Directional (D)	56.1	36.9	48.8	42.0	SN	0.833	0.946	0.833	0.442
							SD	**0.019**	0.090	**0.009**	**0.018**
							ND	**0.002**	0.154	**0.001**	**0.001**

On the left side, the measurement outcomes, EMG at the time of Impact (EMG_(Imp)_), Post-Impact EMG Time *T*_(Post-Imp-Max)_ and Post Impact EMG amplitude EMG_(Post-Imp-Max)_ for retraction muscles and Rebound muscles are listed. The estimated mean from the linear mixed model and the following pairwise analysis tested between the types within the directions are reported. *p*-values are corrected using Benjamini–Hochberg method and *p*-value < 0.05 considered as significant

## Abbreviations

WAD: Whiplash-associated disorder
mTBI: Mild traumatic brain injury
EMG: Electromyography
MVC: Maximum voluntary contraction
T_(Pre-Imp-Onset)_: Time of pre-impact muscle activation onset
EMG_(Pre-Imp-max)_: Maximum EMG amplitude in pre-impact
*T*_(Pre-Imp-Max)_: Maximum EMG time in pre-impact
EMG_(Imp)_: EMG amplitude at impact onset
EMG_(Post-Imp-Max)_: Maximum EMG amplitude in post-impact
*T*_(Post-Imp-Max)_: Maximum EMG Time in post-impact
